# Are women using hormonal contraceptives the risk-takers?

**DOI:** 10.1007/s10654-020-00686-5

**Published:** 2020-09-23

**Authors:** Henning Tiemeier

**Affiliations:** Sumner and Esther Feldberg Chair in Maternal and Child Health, Department of Social and Behavioral Sciences, Harvard TH Chan School of Public Health, 677 Huntington Ave., 6th floor, Boston, MA 02115 USA

More than 20 years ago, I attended an epidemiology course with group assignments on study design. Our group was tasked to design a study of the unintended effects of the third-generation pill. I was the proud presenter. When I returned to my seat I understood my group had failed. Confounding by indication would invalidate our observational design. “It is a difficult challenge”, the instructor consoled us.

The task Hemmingsen et al. have taken on in the present study is much more challenging and is complicated not only by possible confounding by indication [1]. The authors try and conduct a valid study of the offspring neurodevelopmental consequences related to the use of hormonal contraception in the period prior to conception. To this aim they define a nation-wide population-based cohort of more than 1 million children born in Denmark between 1998 and 2014. This cohort is constructed using data from different registries such as the Danish National Prescription Registry to assess contraceptive use, the Danish Patient Register to establish incident ADHD in children, and the Danish National Birth and other registers to obtain demographic and important confounder information. Mothers who recently used contraceptives before or around conception, are about 30% more likely to have a child that will develop ADHD. This pattern of higher offspring ADHD risk was consistently found for all forms of contraception, oral and non-oral, combined and non-combined hormonal contraceptives. The association for progestin-only contraceptives was somewhat stronger; although this effect size difference was clearly not significant. The importance of the present paper is that it raises the possibility that hormonal contraceptive use of the mother might have unintended side effects on the development of the offspring, that is intergenerational effects. Although many unwanted side effects are established, these typically desist relatively soon after the contraceptive use is stopped. Contraceptives are very safe drugs [2]; many researchers and clinicians will find the possibility of intergenerational effects not very plausible.

Luckily, women are not prescribed a certain anti-contraceptive randomly. The choice should be made by the women on the basis of safety (possible unintended effects), effectiveness, and acceptability. However, any of these set of indication criteria may come with certain characteristics of the women and on a group level, that may make a certain unintended outcome more likely. The thromboembolic, cardiovascular and mood problems of the different hormonal contraceptives were difficult for epidemiologists to establish as possible adverse effects [3], a protective association with reproductive cancer such as endometrial cancer is likely but the elevated risk of hormonal contraceptive users to develop breast cancer is still debated [4, 5]. Some hormonal contraceptives, such as the progestin only intra-uterine devices, are clearly understudied in rigorous epidemiological designs [6].

The authors did an excellent job to avoid some typical pitfalls of observational studies. It is hard to argue why a near complete national cohort study in Denmark could suffer from anything but very minimal selection bias. The Danish registries allow a whole country to be followed for the use of medication and for incident diseases such as ADHD. Also, contraceptives are prescribed by the national health care system and not obtained over the counter or in private practice. ADHD was established both by use of medication and diagnosis. There is some argument to be made for each ascertainment method. However, the consistent results suggest that even if there was some underdiagnosis and undertreatment (indeed the prevalence of ADHD was only 2%), misclassification did not introduce substantial bias. Registry data are often characterized by poor control for confounding, but this clearly is a strength of the study. Not only socio-economic status and smoking were included as a covariate but even maternal ADHD. All of these variables are often missing in Scandinavian registry studies [7]. Admittedly, maternal ADHD may be severely underdiagnosed in the present study and this variable is no sufficient control for family history, but the attempt is exceptional.

Are there likely to be unmeasured confounders in the present study? The authors do not show the change of estimate due to different confounders, such as smoking or socio-economic factors, in different models. This common practice demonstrates the extent of confounding that was adjusted for and sometimes helps to estimate the probability of residual confounding by related factors not included in the models. The authors, however, conducted a nice analysis that may help address unmeasured confounding. They showed that some of the associations remained, albeit less strong, if instead of the never users (about 18% of women) past users (70%) served as the reference group. The relatively small minority of women who never use hormonal contraception, may indeed be a more particular group than those that never used the common forms of hormonal contraception. Those that use non-hormonal contraceptive methods or no contraception at all, may have a very different, i.e. lower, risk of neurodevelopmental problems in the offspring.

Residual confounding of the association between hormonal contraceptive use and offspring neurodevelopmental problems, however, remains plausible. Those that used contraception while conceiving or in the last 3 months before may well differ from those that newer used hormonal contraception or stopped longer ago and this difference could be related to the risk of ADHD in the offspring. Hemmingsen et al. describe this possibility but offer no direction that could fulfill this profile. What confounder or set of confounders should we think of? These factors should probably explain the stronger association of non-oral hormonal contraception with offspring ADHD as well. A factor that might be related to smoking, an important confounder in the present study, recent contraceptive users smoked more. As stated above, we know is that the use of contraception is not random. Guidelines and prescription profiles may help a bit. The American Pediatric Association strongly encourages to counsel adolescents to use long-acting reversible contraception (LARC), not necessarily due the few side effects, but because it is long-acting and very effective [8]. Adolescents are considered risk-takers, but such guidelines do not help to sketch a clear user profile.

Even the critical reader of the Danish study may not be convinced that this reasoning likely explains the findings. Perhaps this is because this study is both about confounding by indication and “confounding by discontinuation”. Women also have many reasons why they stop using contraceptives: to conceive, adverse effects, lack of planning to obtain a refill—just to name three. To explain the results, we may wish to argue that the women in the index group, those that become pregnant directly after stopping with hormonal contraceptives or while using them (or at least while still having a prescription), have a higher chance to have ADHD in the offspring than those that never used contraception. ADHD is a highly heritable disease; on a group level, traits of the disorder or other neurodevelopmental symptoms can be found on careful assessment in many parents of children with ADHD. In parents these traits include impulsivity, being disorganized, hot temper, poor planning or risk-taking [9]. But are women with such traits more likely to be prescribed hormonal contraceptives, and more likely to have a LARC prescribed? Would unintentional pregnancies shortly after or while on hormonal contraceptives be more likely in this group? We can only speculate, but it may well be that those prescribed hormonal contraceptives and then discontinuing them, if at all, briefly before conception were slightly more likely to have offspring with ADHD. Or said differently, mothers who themselves may have subtle symptoms of ADHD may prefer the most effective form of contraceptives but discontinue it unintentionally (and perhaps intentionally) more often [10]. Confounding by indication often remains speculation, but that does not make it less common. Doctors and patients, even risk-takers, often make very rational choices.

What next, we need replication of this finding from other Nordic countries and ideally other countries with large registries. A discordant sibling design might help, as recency of contraceptive use may likely differ between pregnancies. I would caution to invest much into exploring potential mechanisms of transgenerational transmission such as epigenetics. This research question will primarily remain an epidemiological challenge for registry studies.

It is certainly too early for doctors and nurses in family planning clinics that offer contraceptive counseling to inform women and their partners about the risk of neurodevelopmental consequences in the offspring. The counselling about the possible side effect of hormonal contraceptive care is often rushed and suboptimal [11]. Moreover, careful personalized information about the possible trade-offs in contraceptive choice, e.g. for smokers, women with a family history of cardiovascular disease or a past mood disorder, is not common practice. In any case, it is premature to warn mothers about this potential long-term impact of contraceptive use in the offspring.

Hemmingsen et al. are to be complimented for addressing an unexpected long-term consequence of hormonal contraceptive use. Epidemiologists must now try to explain the results by confounding, in particular through confounding by indication and confounding by discontinuation. Are women in the index group, those that use contraception shortly prior or during to conception, more likely to have symptoms of ADHD, such as impulsiveness, disorganization, poor planning or risk-taking? Refuting these results, however, will not be easy given the quality of the present study.
